# Synthetic angiotensin II peptide derivatives confer protection against cerebral and severe non-cerebral malaria in murine models

**DOI:** 10.1038/s41598-024-51267-5

**Published:** 2024-02-26

**Authors:** Adriana F. Silva, Marcelo D. T. Torres, Leandro S. Silva, Flavio L. Alves, Antonio Miranda, Vani X. Oliveira, Cesar de la Fuente-Nunez, Ana Acacia S. Pinheiro

**Affiliations:** 1 Centro de Ciências Naturais e Humanas, Universidade Federal do ABC, Santo André, SP Brazil; 2 Escola Paulista de Medicina, Universidade Federal de São Paulo, São Paulo, SP Brazil; 3 Machine Biology Group, Departments of Psychiatry and Microbiology, Institute for Biomedical Informatics, Institute for Translational Medicine and Therapeutics, Perelman School of Medicine, University of Pennsylvania, Philadelphia, PA USA; 4 Departments of Bioengineering and Chemical and Biomolecular Engineering, School of Engineering and Applied Science, University of Pennsylvania, Philadelphia, PA USA; 5 Department of Chemistry, School of Arts and Sciences, University of Pennsylvania, Philadelphia, PA USA; 6 Penn Institute for Computational Science, University of Pennsylvania, Philadelphia, PA USA; 7 Division of Infectious Diseases and Immunology, Department of Medicine, University of Massachusetts Chan Medical School, Worcester, MA USA; 8 Faculdade de Ciências Médicas da Santa Casa de São Paulo, São Paulo, SP Brazil; 9 Instituto de Biofísica Carlos Chagas, Universidade Federal do Rio de Janeiro, Rio de Janeiro, RJ Brazil

**Keywords:** Microbiology, Peptides

## Abstract

Malaria can have severe long-term effects. Even after treatment with antimalarial drugs eliminates the parasite, survivors of cerebral malaria may suffer from irreversible brain damage, leading to cognitive deficits. Angiotensin II, a natural human peptide hormone that regulates blood pressure, has been shown to be active against *Plasmodium* spp., the etiologic agent of malaria. Here, we tested two Ang II derivatives that do not elicit vasoconstriction in mice: VIPF, a linear tetrapeptide, which constitutes part of the hydrophobic portion of Ang II; and Ang II-SS, a disulfide-bridged derivative. The antiplasmodial potential of both peptides was evaluated with two mouse models: an experimental cerebral malaria model and a mouse model of non-cerebral malaria. The latter consisted of BALB/c mice infected with *Plasmodium berghei* ANKA. The peptides had no effect on mean blood pressure and significantly reduced parasitemia in both mouse models. Both peptides reduced the SHIRPA score, an assay used to assess murine health and behavior. However, only the constrained derivative (Ang II-SS), which was also resistant to proteolytic degradation, significantly increased mouse survival. Here, we show that synthetic peptides derived from Ang II are capable of conferring protection against severe manifestations of malaria in mouse models while overcoming the vasoconstrictive side effects of the parent peptide.

## Introduction

Malaria is a human disease caused by unicellular protozoan parasites from the genus *Plasmodium*^1^. As documented by the World Health Organization (WHO) in the latest global malaria report, there were roughly 241 million cases of malaria and 627,000 deaths from malaria worldwide in 2020^2^. The infection has a wide spectrum of symptoms, ranging from “febrile uncomplicated” to severe disease^3^. Severe malaria is commonly associated with *Plasmodium falciparum* infection, and it is considered a life-threatening disease with the occurrence of different pathologies such as severe anemia, respiratory syndrome, cerebral malaria, and malaria associated acute kidney injury^4^. Cerebral malaria is responsible for the highest mortality rates and patients that survive can exhibit long-lasting neurological sequalae, affecting their quality of life^5^.

The mechanism underlying the cerebral damage induced by the malarial parasite has been investigated but has not yet been fully elucidated^6^. The limited tools currently available to avoid or treat neuronal damage calls for new alternative drugs able to eliminate the parasite and prevent irreversible cerebral damage. Currently, the gold-standard treatment for malaria consists of artemisinin-based combination therapies (ACTs); however, increasing levels of parasite-acquired resistance to ACTs has been detected in Southeast Asia^7^. Thus, new strategies to treat severe disease are urgently needed.

Angiotensin II (Ang II), an octapeptide, is considered the main effector of the renin angiotensin system (RAS). It binds and signals through two distinct G protein-coupled receptors (GPCRs): the Ang II type 1 receptor (AT1) and the Ang II type 2 receptor (AT2). By binding to AT1, Ang II induces vasoconstriction, water and sodium retention, and the release of aldosterone, leading to increased blood pressure, cardiovascular remodeling, and fibrosis^8^. In addition to these effects, Ang II has antiplasmodial activity in vitro and in vivo^9–13^. Because of its vasoconstrictor properties, Ang II has been considered for therapeutic use for cardiovascular diseases; its use in cases of vasodilator shock was approved by the U.S. Food and Drug Administration (FDA) in 2017^14,15^. However, its vasoconstrictor activity and high susceptibility to enzymatic degradation (*i.e.*, short half-life) in plasma^16^ are considered major limitations for the widespread use of Ang II as an antimalarial drug.

Here, to overcome the main limitations of the parent peptide, we tested the following synthetic Ang II-derived peptides: VIPF, a linear derivative, which constitutes part of the hydrophobic portion of Ang II; and Ang II-SS, a disulfide bridge-constrained derivative. Both peptides have significant structural differences in relation to the original Ang II peptide and did not elicit significant contractile activity in mouse muscle tissue in an ex vivo assay^11,13^. To test the antiplasmodial activity of these peptides, here we used murine models of severe non-cerebral malaria (BALB/C mice infected with *Plasmodium berghei* ANKA) and experimental cerebral malaria (ECM, C57BL/6 mice infected with *Plasmodium berghei* ANKA). Both peptides reduced parasitemia levels and increased survival in C57BL/6 and BALB/c mice infected with *P. berghei* ANKA. In addition, treatment with VIPF or Ang II-SS reduced the SHIRPA behavioral score when compared to the vehicle group (phosphate buffer saline, PBS). Moreover, we found that Ang II-SS had increased stability as it was more resistant to proteolytic degradation than Ang II. Our results showed that synthetic peptides can overcome the therapeutic limitations of Ang II as an antiplasmodial candidate. These synthetic peptides constitute potential candidates against severe malaria.

## Materials and methods

This study was carried out following the recommendations outlined in the Guide for the Care and Use of Laboratory Animals of the Science and Health Centre. The protocol to assess peptide stability (CEUA-913235) was approved by the ethics committee at Universidade Federal de São Paulo. The animal models protocol (A15-22–008-18) was approved by the ethics committee of Universidade Federal do Rio de Janeiro.

### Synthetic peptides and other reagents

The peptides used in this study were synthesized using the Wang-resin^17^ acquired from Advanced Chemtech. The N-^α^Fluorenylmethyloxicarbony-L-amino acids (Fmoc-aa) were acquired from AAPPTECH and Bachem Inc. The Fmoc-Phe-Wang-resin with replacement degree 0.90 mmol g^−1^ was used for the linear peptides while the Fmoc-Cys(Trt)-Wang-resin (0.55 mmol g^−1^) was used for the constrained peptide. The amino acid derivatives with protected side chains used in the synthesis were: Cys(Trt), His(Trt), Tyr(tBu), Arg(Pfb), Asp(OtBu). Reagents and solvents purchased from Merck and Sigma-Aldrich were utilized for analytical and chromatographic experiments.

### Peptide synthesis, characterization, and purification

The linear and constrained peptides were synthesized by the solid-phase peptide synthesis (SPPS) method using the Fmoc strategy^18^. We used Fmoc-aa-hydroxymethylated resin as solid support^19^ with substitution degrees ranging from 0.6 to 1.00 mmol g^-1^. Couplings were performed from 1 to 2 h, with 2.5 of excess of N,N'-Diisopropylcarbodiimide/1-Hydroxybenzotriazole (DIC/HOBt) in dichloromethane/dimethylformamide (DCM/DMF; 1:1; v/v), and coupling was monitored by the Kaiser test reaction^20^. The peptides were deprotected and cleaved from the resin in a reaction with 90% trifluoroacetic acid (TFA), 5% water, and 5% of the scavenger solution (anisole or thioanisole), so that the concentration of peptidyl-resin was 10 mL g^-1^. The cleavage reaction was 2 h long at room temperature. At the end of the cleavage reaction, the peptide and resin were washed with diethyl ether (distilled and stored in a freezer at − 24 °C), and the peptide was extracted from the resin with a 0.1% TFA in 90% of ACN/H_2_O and lyophilized.

The constrained peptide’s disulfide bridge in peptide Ang II-SS (**CDRVYIHPFC**, where underlined residues are part of the cyclization, *i.e.*, all residues of the peptide sequence) was formed before the purification of the peptide and after the cleavage, deprotection, and lyophilization steps. The peptide was dissolved in 80% acetic acid/water (AcOH/H_2_O) solution (peptide concentration of 1 mg mL^−1^). After the addition of 0.04 mol L^−1^ of iodine (I_2_), the resulting solution was stirred for 40 min at room temperature^21^; diethyl ether was added to the solution, the peptide was extracted with water and frozen, and the aqueous solution was lyophilized.

The peptides were purified in a semi-preparative Waters liquid chromatography system, coupled to a UV–Vis detector—model 2489. The solvents used were HPLC grade. Water was obtained from a Milli-Q system, from Millipore, equipped with cartridges for the retention of salts and organic compounds. The system was controlled by a workstation by using the Empower software. The experimental conditions used were: Phenomenex C_18_ column (21.2 mm × 250 mm, 300 Å, 15 μm), solvent system: A: 0.1% TFA/H_2_O, B: 0.1% TFA in 90% ACN/H_2_O, gradient: 0.33% of B min^−1^, flow: 10 mL min^−1^, and wavelength: 220 nm.

The peptides were characterized (Table 1) by liquid-chromatography electrospray-ionization mass spectrometry (LC/ESI–MS) using a Mass Spectrophotometer model 6130 Infinity coupled to a Model 1260 HPLC system (Agilent) and an automatic injector with capacity for 120 samples. The experimental conditions used were Phenomenex Gemini C_18_ column (2.0 mm × 150 mm, 110 Å, 3.0 μm), solvent system: A: 0.1% TFA/H_2_O, B: 0.1% TFA in 90% ACN/H_2_O, gradient: 5–95% of B in 20 min, flow: 0.2 mL min^−1^, wavelength: 220 nm, injection volume: 20 μL, and mass range: 100–2500 Daltons.

**Table 1:** Peptide information.

Peptide	Sequence	HPLC purity^a^ (%)	Molecular weight (MW)	Obtained molecular weight (m + H^+^)^b^
Ang II	DRVYIHPF	98	1045.6	1046
Ang II-SS	**CDRVYIHPFC**	98	1247.6	1248
VIPF	VIPF	99	474.6	475

Sequences, purity, molecular weight, and mass spectrometry data of peptides used in this study.

^a^The HPLC profiles were obtained under the following conditions: Column: Supelcosil C_18_ (4.6 × 150 mm), 60 Å, 5 μm; Solvent system: A: 0.1% TFA/H_2_O and B: 0.1% TFA in 60% ACN/H_2_O; gradient: 5–95% solvent B in 30 min; flow: 1.0 mL min^−1^; λ = 220 nm; injection volume: 50 µL and sample concentration: 1.0 mg mL^−1^.

^b^The LC/ESI–MS profiles were obtained under the following conditions: Column: Phenomenex Gemini C_18_ (2.0 × 150 mm),110 Å, 3.0 μm; Solvent system: A: 0.1% TFA/H_2_O and B: 0.1% TFA in 90% ACN/H_2_O; Gradient: 5–95% of B in 20 min; Flow: 0.2 mL min^−1^; λ = 220 nm; Injection volume: 20 μL; Sample concentration: 1.0 mg mL^−1^ and Mass range: 100–2,500 Daltons.

The bold sequence (Ang II-SS) is to indicate that all residues of the sequence are part of the disulfide bridge formed between the two cysteine residues at each terminus of the peptide.

### Proteolytic stability of peptides in mouse blood plasma

Peptide stability in plasma was evaluated using a pool of blood plasma from healthy mouse donors. An aliquot of 20 µL peptide, at a concentration of 10 mg mL^−1^, was added to 1 mL of 25% fresh plasma in phosphate-buffered saline (PBS) and incubated at 37 °C in triplicate for time intervals of 0, 5, 15, 30, 60, 120, 240, and 360 min. During incubation, 100 µL of sample were withdrawn and incubated with 10 µL of pure TFA at 5 °C for 15 min. The resulting mixtures were centrifuged at 300 × *g* for 5 min. A volume of 30 µL of the supernatants was injected in an online HPLC coupled to a mass spectrometer (LC/ESI–MS) using a Mass Spectrophotometer model 3100 Waters coupled to a Model 2690 HPLC system (Waters). Each peptide was identified by mass spectrometry, and the respective molecular weight was verified. The experimental conditions applied were Luna C_18_ column (2.0 mm × 150 mm), 100 Å, 3.0 μm, solvent system: A: 0.1% TFA/H_2_O, B: 0,1% TFA in 60% ACN/H_2_O, gradient: 5–95% of B in 30 min, flow: 0.2 mL min^−1^, wavelength: 220 nm, injection volume: 20 μL, and mass range: 100–2000 Daltons.

### In vivo infection with *P. berghei* ANKA

Six- to eight-week-old male C57BL/6 mice were provided by the Oswaldo Cruz Foundation (FIOCRUZ—Rio de Janeiro, Brazil). The mice were housed in appropriate cages with free access to food and fresh water in a room with controlled temperature (22–24 °C) with a light/dark cycle of 12 h.

A cryopreserved sample of erythrocytes infected *with P. berghei* ANKA (kindly provided by Dr. Leonardo J. Carvalho, La Jolla University, San Diego, CA) was thawed and 1 × 10^6^ erythrocytes infected with *P. berghei* ANKA were injected intraperitoneally (i.p.) in the C57BL/6 mice. Parasitemia was monitored from the prepatent period until parasitemia was sufficient for infection of the experimental animal groups (7 male mice per group), i.e., 5–8% parasitemia per mL of blood (injection of 200 µL in each mouse).

The same protocol was performed with 6- to 8-week-old BALB/c mice also provided from FIOCRUZ (8 male mice per group). Each group was treated one hour before infection (day zero) i.p. at 100 ng Kg^−1^ for five consecutive days. The dose used was similar to that used for Ang II in in vivo studies, as described by Gallego-Delgado et al., 2015. Infected and untreated mice were used as a control. After 3 days, parasitemia was evaluated by blood smear from a droplet of blood collected from the lateral tail vein of each animal and colored with the hematological Diff-Quick^®^ kit. The survival of the animals was also monitored daily. Survival analyses were performed by the Kaplan–Meier log rank test.

### Behavioral tests

C57BL/6 mice were used for the experimental cerebral malaria model, and the assays were defined by the presence of at least two of the following clinical signs of neurological involvement: ataxia, paralysis of the limbs, poor correction reflex, convulsions, and coma. A series of SHIRPA tests *(****S****mithKline Beecham, ****H****arwell, ****I****mperial College,* **R***oyal London Hospital, ****p****henotype ****a****ssessment)* was used to evaluate behavioral, motor, and neurological changes during the development of the *P. berghei* infection^22^*.* Tests aimed at evaluating cognitive and behavioral parameters were selected to assess brain impairment of the mice. The tests described below were initially performed on animals not yet infected at day 0. Next, the mice were treated and infected as described earlier. Five days after infection, the tests were repeated and the results were expressed as the difference between the sum of the scores before and after infection with *P. berghei*^23^. Lower scores (close to zero) represent the maximum performance (or least impairment), while higher scores (close to 20) represent severe behavioral impairment. The behavioral tests contributing to the score were as follows:

#### Behavior recorded in the arena

Initially, the animal was placed inside a conical structure of glass, located on a white platform*.* The following aspects were observed: body position, spontaneous activity, breathing rate, tremor, number of headings (support only on the hind legs), and grooming (movements of “cleaning” directed to the head or body, performed with the forelegs) of the animals.

#### Transfer excitement

After five minutes of the test described above, the animals were transferred to the arena without being touched. The observation of the immediate reactions to the new environment was recorded for thirty seconds and a score was assigned.

#### Locomotor activity

While mice were still in the arena, the number of squares of 11 cm^2^ of the arena that the animal stepped into with four legs at the same time was quantified for thirty seconds.

#### Tail elevation

During the evaluation of the locomotor activity, the level of elevation of the animal’s tail was also observed. Mice were scored from 0 to 3, in which “3” corresponded to dragging the tail on the ground and “0” corresponded to hightail (above 45°).

#### Behavior recorded above the arena

The animals were removed from the arena by the tail and their movements and ability to loosen up were observed, at which point they were placed on top of the grid.

#### Grid maneuver

The animals were held above the grille by the suspension of the tail so that they managed to pick up the grille with their front paws. Then the animals were kept stretched and rotated horizontally until the body was above the grid and then the animal was released. The animal’s reaction was recorded through a score in which “4” meant that the animals fell immediately upon being released and “0” meant that they actively grabbed the grille with the hind legs (as well as the forelegs).

#### Behavior recorded during supine restriction

The animals were contained in a supine position and visual positioning capacity, grip strength, abdominal tone, and pinched reflex on the hind paw fingers were observed. Also, at that moment the length of the animals was measured from the tip of their nose to the base of the tail.

#### Contact postural correction reflex

The animals were placed inside a tube four centimeters in diameter and turned upside down. The ability of the animals to turn and stand upside down or not was recorded and scored as “1” for missing the capacity and “0” for having the capacity.

### Blood pressure measurement

At day 5 post-infection, 24 h after the last injection of peptides, the mean arterial blood pressure of the animals was evaluated noninvasively using the tail-cuff method, described by Kubota et al.^24^. Briefly, the mice were immobilized in a plastic restrainer appropriate to their size, then placed on a tail plethysmograph, and then preheated in a chamber at 35 °C for 10 min. A device with a pneumatic pulse sensor (cuff) was attached to the tail. The mice were conditioned in this environment (to get used to the procedure) for 7 days before readings were performed.

Blood pressure, expressed in millimeters of mercury (mm Hg) and heart rate values were recorded in an MK-200 model device (with heating) and were calculated, on average, from at least three consecutive readings obtained for each mouse.

### Statistical analyses

The data were analyzed by one-way (stability and vasoconstrictive activity) or two-way unidirectional (mouse experiments) analysis of variance (ANOVA). To evaluate the statistical significance of the differences between the groups the Kruskal–Wallis or Turkey comparison test was used, as described in the figure legend. The data are expressed as mean ± standard deviation and differences between groups were considered statistically significant when p < 0.05. The statistical significances are represented in the figure legend as: *P < 0.05, **P < 0.01, ***P < 0.001, ****P < 0.0001. The percentage of surviving mice graphics was plotted as a Kaplan–Meier curve. Treated groups were compared to the vehicle group by log-rank (Mantel-Cox) test and were considered statistically significant when p < 0.05. Analysis was performed on GraphPad Prism software – version 9.0 (GraphPad Software, San Diego, California, USA) (www.graphpad.com). In the in vivo experiments, we used 7 mice per group.

### Ethical statement

All animal procedures were carried out in accordance with the Guide for the Care and Use of Laboratory Animals of the National Institutes of Health in the United States of America. All experimental protocols were previously submitted and approved by the Institutional Ethics Committee of Federal University of Rio de Janeiro (protocol number A15-22-008-18). The study is reported in accordance with ARRIVE guidelines.

## Results

### Peptide synthesis and proteolytic stability in mouse serum

All peptides were synthesized manually by SPPS using the Fmoc strategy and purified by HPLC. The peptides had a purity of > 98% and the obtained molecular weight was very close to the molecular weight predicted for each peptide (Table 1).

**Figure 1:**
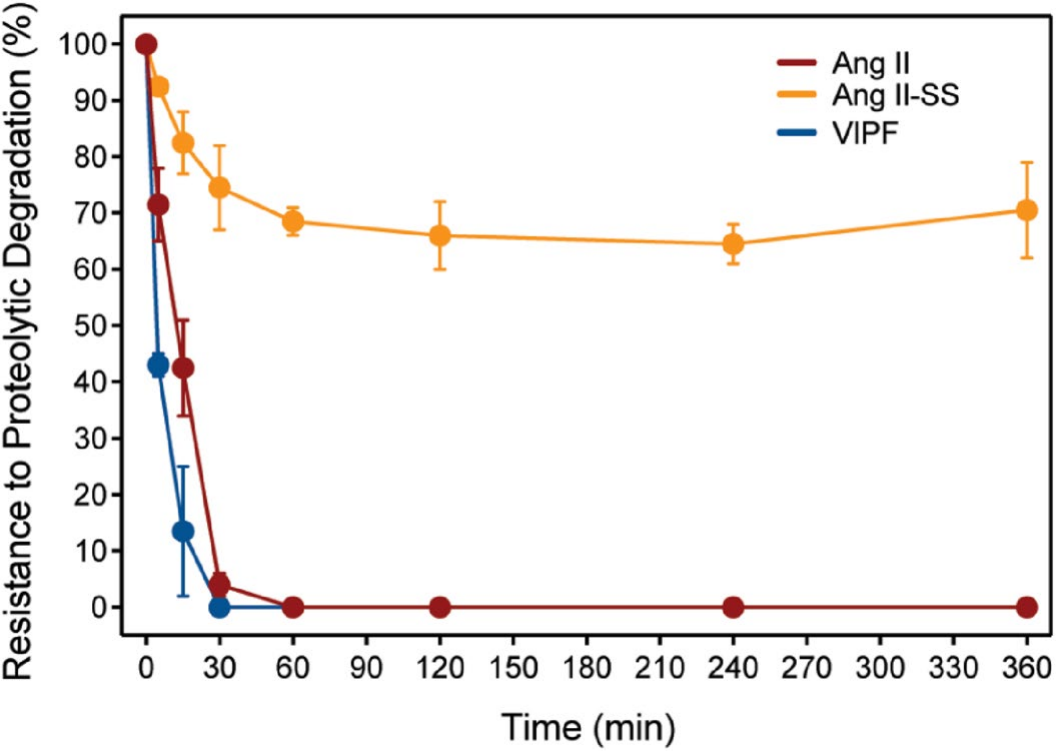
Proteolytic stability of peptides in mouse serum. Proteolytic stability of Ang II (red line), Ang II-SS (orange line), and VIPF (blue line) peptides was assessed by interaction with proteases from fresh mouse serum at time points of 0.5, 1, 2, 4, and 6 h. Aliquots were analyzed by liquid chromatography-electrospray ionization-mass spectrometry (LC/ESI–MS) and values corresponding to percentage of the area under the peak were normalized based on the initial dose of each peptide. Data are representative of two independent experiments and are shown as mean ± SD, n = 9, as determined by one-way ANOVA followed by Kruskal–Wallis’ comparison test.

Stability assays of Ang II and its derivatives were performed using freshly extracted mouse serum. Peptide aliquots in the presence of serum were collected and analyzed by LC–MS/ESI for 6 h. The values shown correspond to % of the area under the peak normalized according to the initial concentration of each peptide. All experiments were performed in duplicate (Fig. 1). The constrained derivative Ang II-SS was stable for a remarkably extended period (70% remaining after 6 h of experiment), whereas Ang II and VIPF were completely degraded after 30 min.

### Ang II-derived peptides lacked vasoconstrictive effects in mice

**Figure 2:**
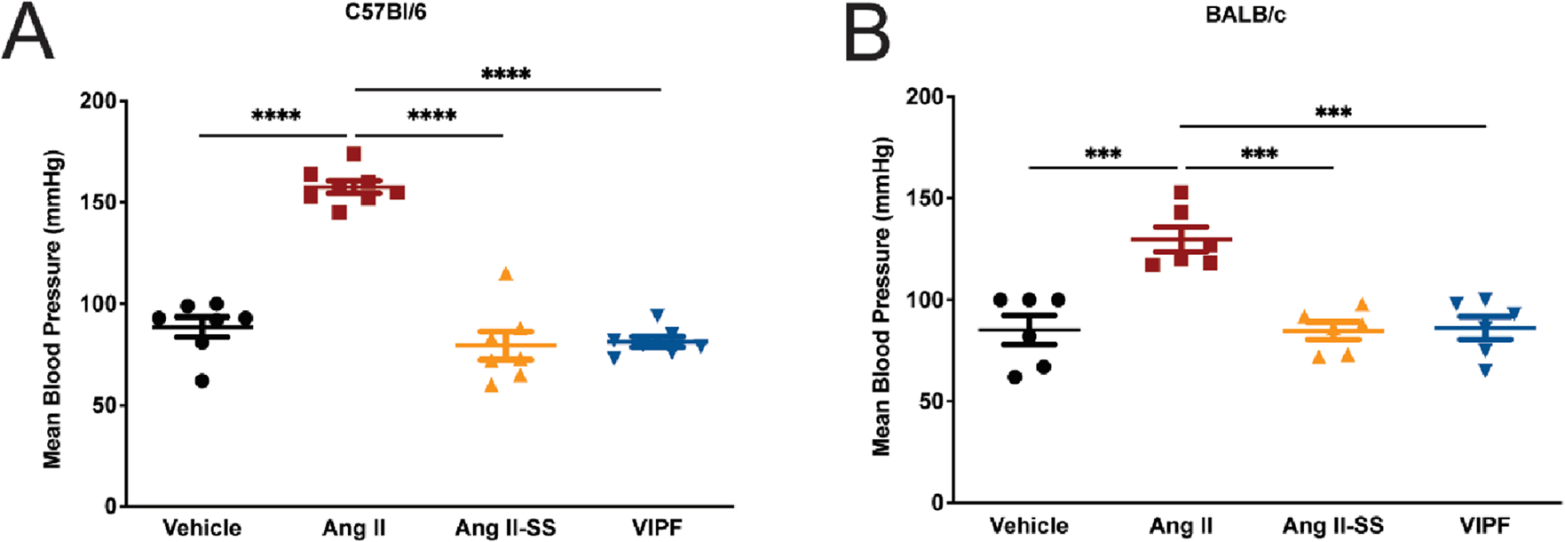
Ang II-derived synthetic peptides lacked vasoconstrictive effects in mice. The mean arterial blood pressure of **(A) **C57BL/6 mice (n = 7, except for the Ang II group where n = 8) and **(B)** BALB/c mice (n = 6) treated with Ang II (red square), Ang II-SS (orange triangle), VIPF (blue inverted triangle), or vehicle (PBS, black closed circle) was evaluated at day 5 post-infection using a tail-cuff method. Data are shown as mean ± SEM from a single experiment. ***P < 0.001, ****P < 0.0001, as determined by one-way ANOVA followed by Tukey’s multiple comparisons test compared to vehicle (black closed circle), n = 7.

Although the antiplasmodial activity of Ang II has been demonstrated, the major limitation for its use as an antimalarial agent is its vasoconstrictor activity since Ang II causes vasoconstriction by binding to the active site of the AT1 receptor. Therefore, we tested the effect on mean blood pressure of the two synthetic Ang II-derived peptides, VIPF and Ang II-SS, by the tail-cuff method in both C57BL/6 mice and BALB/c mice. Five daily doses (100 ng Kg^−1^ min^−1^) of peptides were given at day 5 post-infection. Ang II treatment increased the mean blood pressure by about 60% in both C57BL/6 and BALB/c mice, but no changes were observed in infected mice treated with either of the synthetic peptides, as the treated mice maintained mean blood pressure values similar to those of the vehicle control group (Fig. 2A and B).

### Ang II-derived synthetic peptides reduced parasitemia and increased survival in experimental cerebral malaria and severe non-cerebral malaria mouse models

**Figure 3:**
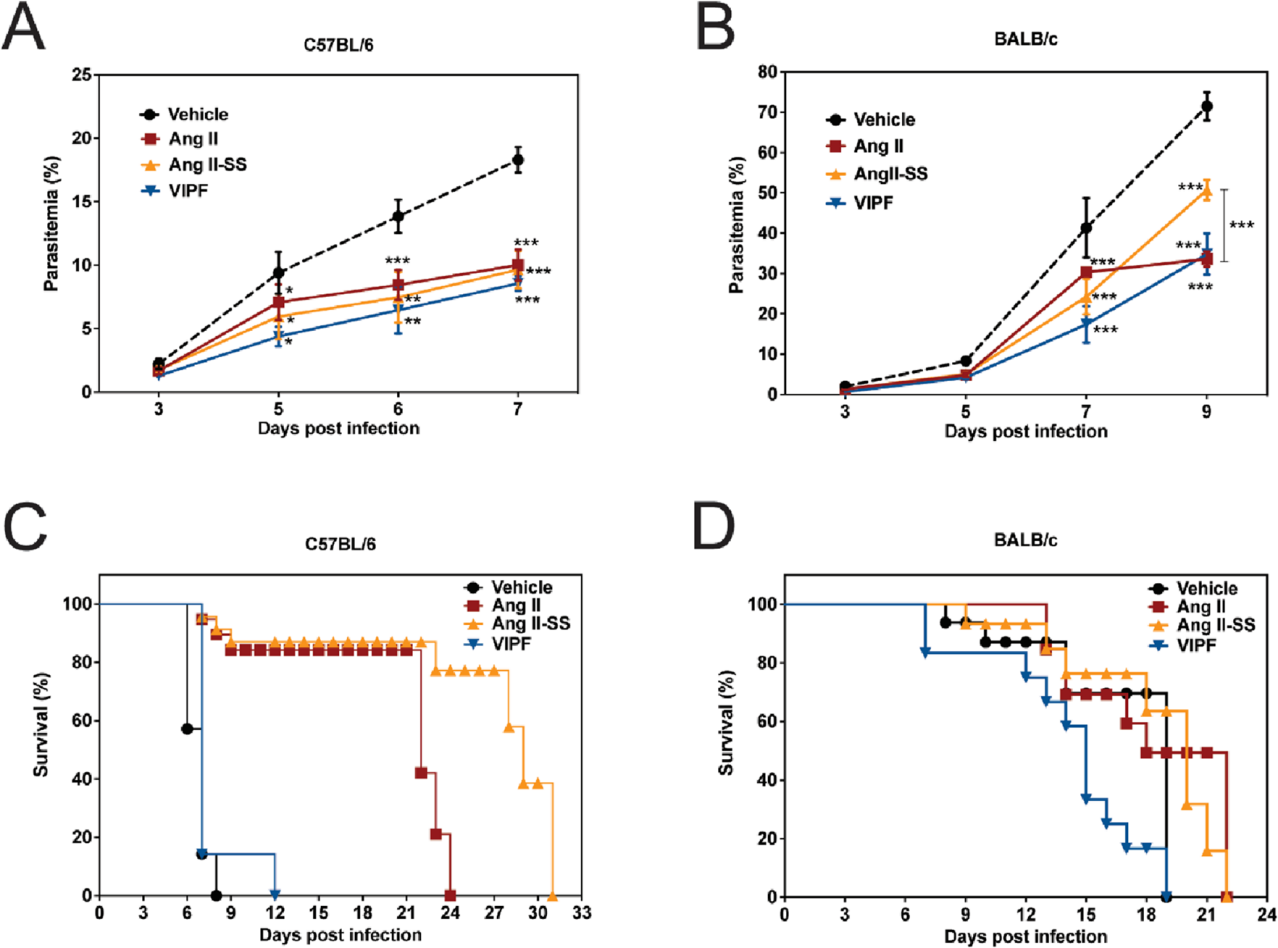
Ang II-derived synthetic peptides reduced parasitemia and Ang II-SS increased survival in experimental cerebral malaria and severe non-cerebral malaria mouse models. C57BL/6 (cerebral malaria model) and BALB/c (non-cerebral malaria model) mice received a single daily dose (100 ng Kg^−1^ min^−1^) of Ang II (brown line), Ang II-SS (orange line), VIPF (blue line), or vehicle (PBS, black line) for 5 days. The initial dose was injected one hour before mice were infected with *P. berghei* ANKA. Parasitemia was monitored by Diff-Quick stained blood smears from day 3 to day 7 for C57BL/6 mice (**A**) and from day 3 to day 9 for BALB/c mice (**B**). Percentage of survival for C57BL/6 mice (**C**) and BALB/c mice (**D**) mice was monitored daily starting on day 6 p.i. Data in A and B are shown as mean ± SD from a single experiment. *P < 0.05, **P < 0.01, ***P < 0.001, as determined by two-way ANOVA followed by Tukey’s multiple comparisons test compared to vehicle (black asterisks) or Ang II-SS (orange asterisks), n = 8. Data in C and D are shown as Kaplan–Meier plots of the percentage of surviving mice. Treated groups were compared to the vehicle group by log rank (Mantel-Cox) test, n = 8.

The antimalarial properties of the Ang II-derived synthetic peptides were tested in vivo in two models: (1) experimental cerebral malaria (ECM) established in C57BL/6 mice infected with *P. berghei* ANKA; and (2) a severe non-cerebral malaria mouse model obtained by infecting BALB/c mice with the same parasite strain. For each mouse strain (C57BL/6 and BALB/c), three groups of naïve mice received a single intraperitoneal dose of Ang II or Ang II-derived peptides (100 ng Kg^−1^ min^−1^), administered daily until day 4 post-infection (p.i.). A fourth control group received the same volume of the vehicle (1X PBS) (Supplementary Fig. 1). Parasitemia was accessed by thick blood smear from day 3 until day 7 p.i. for C57BL/6 mice and until day 9 p.i. for BALB/c mice. When infected C57BL/6 mice were treated with Ang II, VIPF, or Ang II-SS, we observed a reduction in parasitemia levels to approximately 10% of the original level at day 7 p.i., when compared to the vehicle control group, which reached 18% parasitemia (Fig. 3A). A reduction in parasitemia was also observed in BALB/c mice groups (Fig. 3B). Although both VIPF and Ang II-SS reduced parasitemia in infected BALB/c mice, especially at day 9 p.i., VIPF had a stronger anti-plasmodial effect than Ang II-SS. In both models, treatment with Ang II-SS led to increased mouse survival. Importantly, in the ECM model, mice died outside the window characteristic of cerebral malaria, suggesting Ang II-SS’s potential therapeutic use in combating cerebral malaria (Fig. 3C and D).

### Mice treated with Ang II-derived synthetic peptides had low predictive score indicating small likelihood of developing ECM

**Figure 4:**
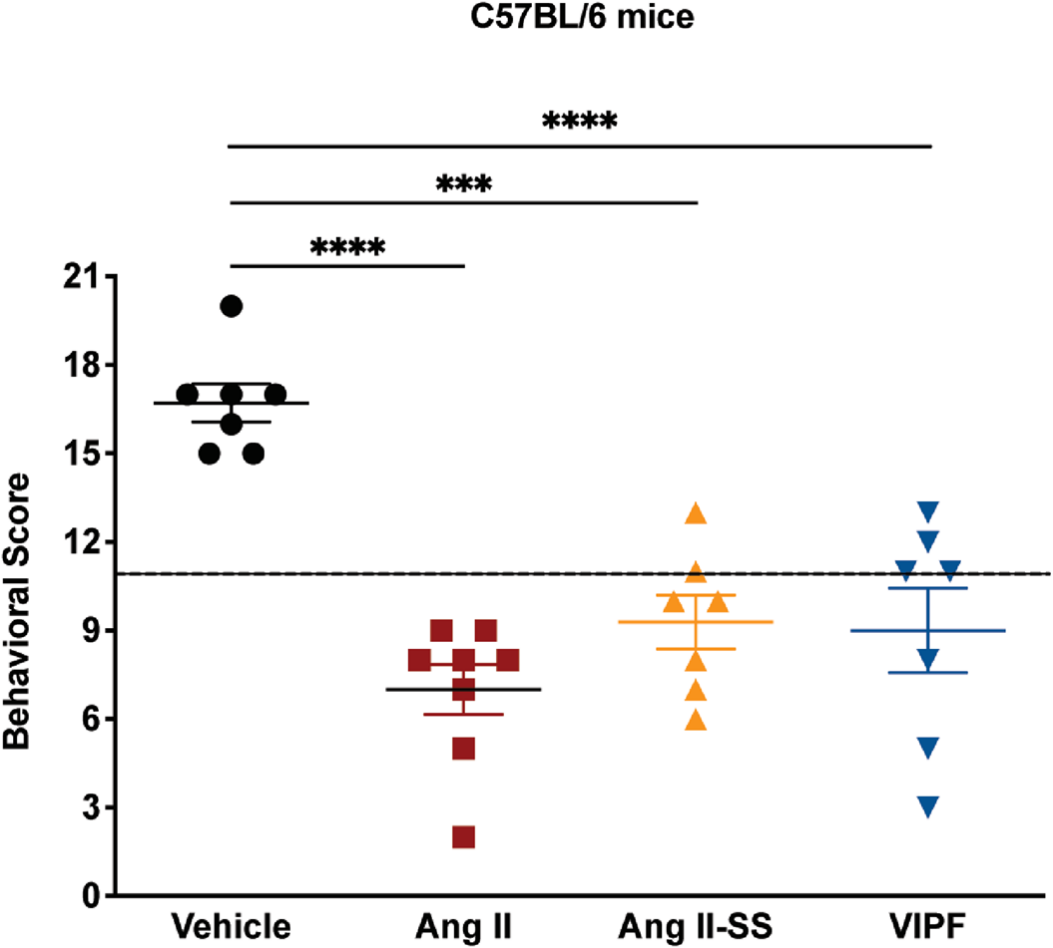
Mice treated with Ang II and its synthetic peptide derivatives had low predictive score indicating low probability of developing ECM. Behavioral and functional analysis was performed 5 days post-infection in *P. berghei* ANKA-infected C57BL/6 mice treated with Ang II (red square), Ang II-SS (orange triangle), VIPF (blue inverted triangle), and the vehicle control group (black closed circle). The results are expressed as differences between values recorded before the infection and 5 days post-infection. Data are shown as mean ± SEM from a single experiment. ***P < 0.001, ****P < 0.0001, as determined by one-way ANOVA followed by Bonferroni’s multiple comparisons test compared to the vehicle control group, n = 7, except for the Ang II group where n = 8.

The probability of C57BL/6 mice infected with *P. berghei* ANKA to develop ECM can be quantified by the SHIRPA protocol (SmithKline, Harwell, Imperial College, Royal Hospital, Phenotype Assessment)^22^. The adapted SHIRPA protocol was performed at day 5 post-infection in all four mouse groups as previously described^23^. Both Ang II-derived peptides, as well as Ang II, showed reduced SHIRPA scores when compared to the vehicle control group (Fig. 4). No difference in the animal body weight change was observed at day 5 p.i. when compared to the untreated control group (Fig. S2).

## Discussion

Ang II, a peptide hormone derived from RAS, has been proposed to impair the life cycle of *Plasmodium* spp., as demonstrated in several models^9–13^. However, the development of Ang II as an anti-malarial drug has been limited by its vasoconstrictive effects. Here, we tested Ang II-derived peptides for their ability to overcome both the susceptibility of Ang II to proteolytic degradation and its classical AT1-mediated vasoconstriction. Using ECM and a non-cerebral severe malaria mouse model obtained by infecting BALB/c mice with *P. berghei* ANKA, we confirmed the antiplasmodial effect of the synthetic Ang II-derived peptide, Ang II-SS. Our results revealed Ang II-SS as a potential therapeutic candidate against the development of severe malaria.

Mouse models have been extensively used as a tool to understand the pathogenesis of severe disease in humans. ECM differs from human cerebral malaria in certain respects but preserves common features, such as the accumulation of infected erythrocytes in the brain vasculature and the occlusion of brain capillaries^25^. On the other hand, the infection of BALB/c mice *with P. berghei* ANKA does not produce cerebral disease but does lead to severe malaria and death, especially resulting from severe anemia^26^. Thus, mouse models can be useful in understanding the development of malaria and the different disease outcomes. Here, we used ECM and BALB/c murine models to test the ability of Ang II-derived peptides to impede the progression of malarial disease in vivo.

Gallego-Delgado et al.^12^ used ECM to demonstrate that Ang II reduced parasitemia after 7 days of infection and delayed the development of ECM, while also leading to increased mouse survival. Because Ang II is quickly degraded, intradermal mini pumps were used to maintain constant blood levels of Ang II at high concentrations without affecting blood pressure in mice. In the same mouse model, a discrete but significant reduction in parasitemia was observed when infected mice were treated with captopril, an ACE inhibitor used to reduce the formation of Ang II^27^. In this study, the two Ang II-derived peptides, VIPF and Ang II-SS, were selected because they: (1) reduced parasitemia in several *Plasmodium* species^11^; (2) lacked vasoconstrictor activity in vitro^11,13^; and (3) were more resistant to proteolytic degradation in plasma compared to Ang II^13^.

Although the three peptides tested were similarly able to reduce parasitemia levels, the best effect in increasing survival was detected for mouse groups treated with Ang II-SS. We hypothesize that this result is caused by VIPF’s higher susceptibility to proteolytic degradation compared to both Ang II, and particularly compared to Ang II-SS (Fig. 1). Ang II-SS has a head-to-tail peptide cyclization formed by a disulfide bridge^28^, which increased its stability and likely also contributes to the observed increased mouse survival.

It is worth noting that VIPF treatment reduced parasitemia in a cerebral malaria model after 7 days (Fig. 3A). This timepoint approximately coincides with the slight protection conferred by VIPF in mice (Fig. 3C). Therefore, these data correlate reduced parasitemia effects with increased survival in mice for VIPF treatment. The behavioral assays (Fig. 4) show a significant reduction in predictive score upon VIPF treatment after 5 days, which is within the timeline of observed: (1) decreased parasitemia (Fig. 3A); and (2) slight protection (Fig. 3C). It is important to keep in mind that the behavioral score (SHIRPA assay) itself does not rule out the occurrence of cerebral disease. Moreover, mice die very quickly, typically between days 6 and 12, in the ECM model used in our work (Fig. 3C). This is a time window characteristic of cerebral disease occurrence. We also hypothesize that other factors, such as potential immunomodulatory activities of the peptides, may contribute to the observed results in the ECM model.

Since all three peptides reduced parasitemia levels to the same extent, it is plausible that both Ang II and Ang II-SS could also exert activity through unrelated and independent effects, presumably specific to the pathogenesis of cerebral malaria. It is important to highlight that survival depends not only on the level of parasitemia but also on a combination of host and parasite factors, including a complex host immune response, in addition to potential immunomodulatory effects of the peptides. The fact that we did not observe vasoconstrictor activity in vitro suggests that the synthetic peptides likely do not bind and signal via the AT1 receptor. A previous study demonstrated that blockage of the AT1 receptor or stimulation of AT2 prevented brain damage and increased survival while AT2 receptor knockout mice were more susceptible to cerebral malaria^29^. We observed that Ang II-SS protected against cerebral malaria, but further experiments are needed to clarify its mechanism of action.

In summary, we report the in vivo antimalarial effects of Ang II-derived synthetic peptides and provide data pointing to a lack of vasoconstrictor effect of these peptides, overcoming one of the major limitations of Ang II as an antimalarial drug. More broadly, the results reported here support the exploration of naturally occurring peptides as basic scaffolds for the development of potential new therapies for a wide range of infections.

## Conclusions

We have shown that the synthetic peptide Ang II-SS (**CDRVYIHPFC**) displays increased proteolytic resistance and non-vasoconstrictor activity compared to its parent peptide, Ang II. These results provide evidence that overcomes the main limitations for developing Ang II as an antimalarial drug. Ang II-SS exhibited antiplasmodial activity against the blood stage of the malarial parasite and significantly increased mouse survival in relevant models. Overall, our results show that synthetic peptides derived from Ang II represent potential therapeutic candidates against severe malaria.

### Supplementary Information

Supplementary Figures.

## Data Availability

The datasets used and/or analyzed during the current study available from the corresponding author on reasonable request.
