# Supplementary material for: Synthetic angiotensin II peptide derivatives confer protection against cerebral and severe non-cerebral malaria in murine models
Source: Sci Rep. 2024 Feb 26;14:4682. doi: 10.1038/s41598-024-51267-5 (PMC10897374; doi:10.1038/s41598-024-51267-5)
Supplement: Supplementary file 1 — Supplementary Figures. [file 41598_2024_51267_MOESM1_ESM.docx]

**Synthetic angiotensin II peptide derivatives confer protection against cerebral and severe non-cerebral malaria in murine models**

Adriana F. Silva^1,2^, Marcelo D. T. Torres^3-5^, Leandro S. Silva^6^, Flavio L. Alves^2^, Antonio Miranda^2^, Vani X. Oliveira Jr^1,2^, Cesar de la Fuente-Nunez^3-5,*^ and Ana Acacia S. Pinheiro^7,*^

**Supplementary Information file**

Supplementary Figures 1 to 2

**Supplementary Figures**

**
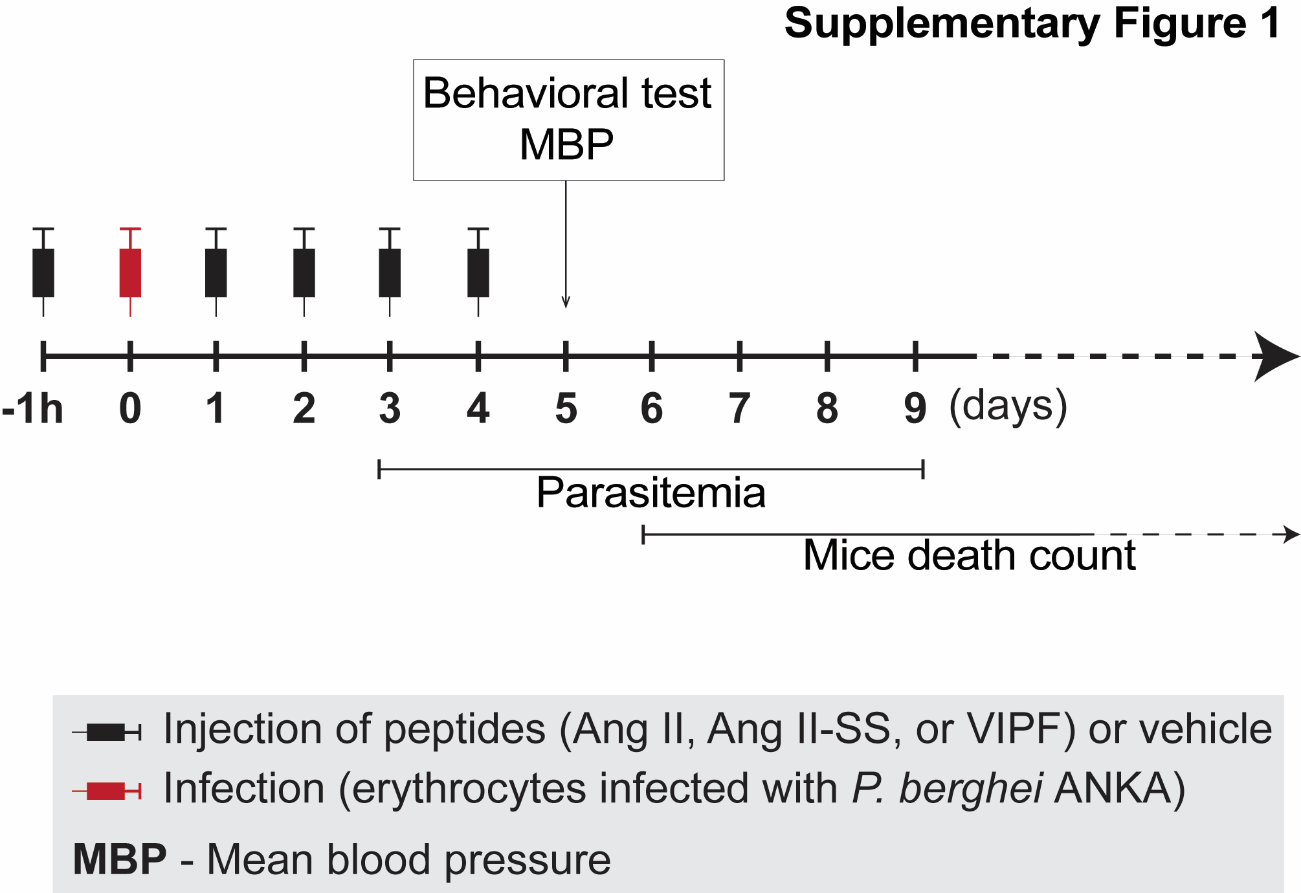
**

**Supplementary Figure 1. Experimental design showing peptide injection and infection in a mouse model.** C57BL/6 and BALB/c mice were allocated to three groups, which received a single intraperitoneal dose of Ang II, VIPF, or Ang II-SS (100 ng Kg^-1^ min^-1^) daily until day 4 post-infection (p.i.). A fourth control group received the same volume of 1X PBS (vehicle). First injections of peptides or vehicle were performed one hour before infection. All mice were infected with 10^6^ *P. berghei* ANKA-infected erythrocytes in 200 µL of 1X PBS. Parasitemia was assessed by a thick blood smear from day 3 until day 7 p.i. for C57BL/6 mice and until day 9 p.i. for BALB/c mice. Behavioral tests and mean blood pressure (MBP) were assessed at day 5 p.i. and the number of dead mice was counted from day 6 p.i. until the end of the experiment.

**
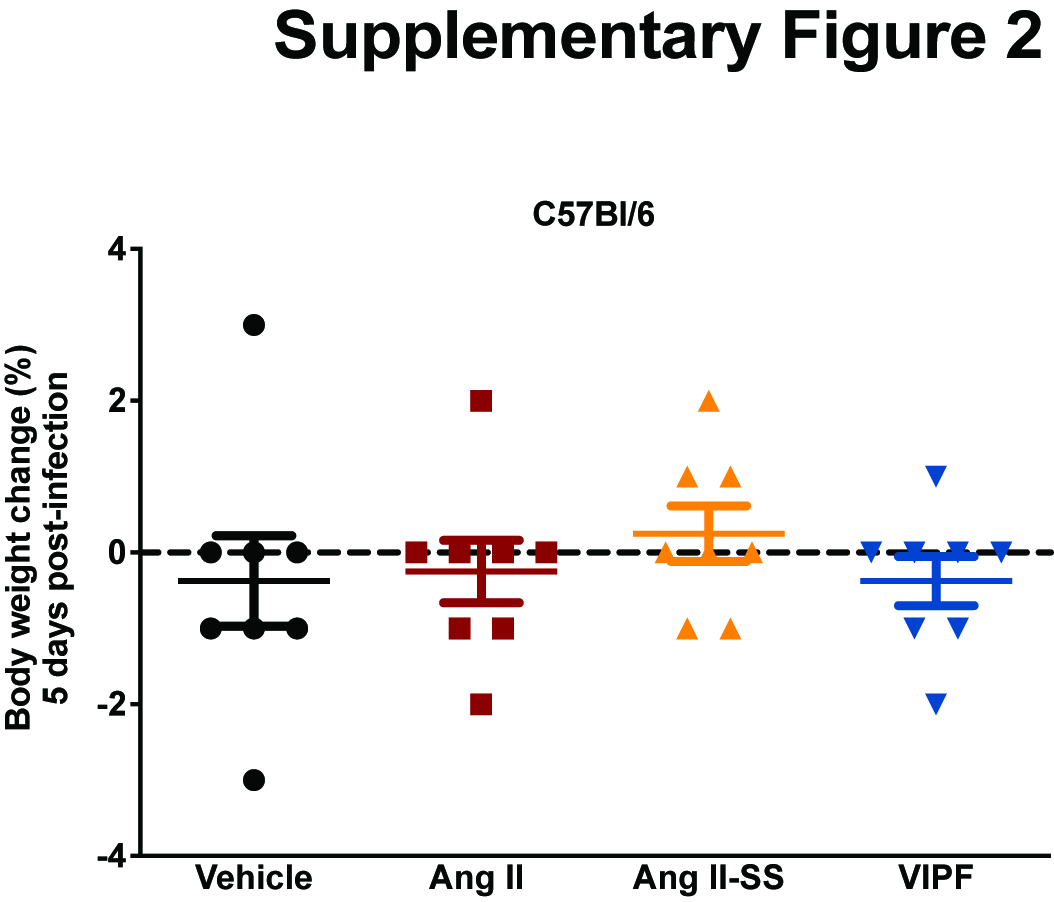
**

**Supplementary Figure 2. Mouse body weight monitoring.** C57BL/6 mice treated with Ang II, VIPF, or Ang II-SS were weighed at day zero and five days after *P. berghei* infection. Data are expressed as percentage of body weight change. Data are shown as mean ± SEM from a single experiment. Comparison between groups was assessed by one-way ANOVA followed by Tukey’s multiple comparisons test compared to vehicle, n = 8.
